# Deep learning reconstruction for accelerated 3-D magnetic resonance cholangiopancreatography

**DOI:** 10.1007/s11547-025-01987-z

**Published:** 2025-03-18

**Authors:** Jan M. Brendel, Reza Dehdab, Judith Herrmann, Stephan Ursprung, Sebastian Werner, Haidara Almansour, Elisabeth Weiland, Dominik Nickel, Konstantin Nikolaou, Saif Afat, Sebastian Gassenmaier

**Affiliations:** 1 Department of Radiology, Diagnostic and Interventional Radiology, Tuebingen University Hospital, University of Tuebingen, Hoppe-Seyler-Str. 3, 72076 Tuebingen, Germany; 2 MR Application Predevelopment, Siemens Healthineers, Forchheim, Germany; 3 Cluster of Excellence iFIT (EXC 2180) “Image-Guided and Functionally Instructed Tumor Therapies”, University of Tübingen, 72076 Tuebingen, Germany

**Keywords:** Deep learning, MRI, Acquisition time, Biliary, Pancreas

## Abstract

**Purpose:**

This study aimed to compare a conventional three-dimensional (3-D) magnetic resonance cholangiopancreatography (MRCP) sequence with a deep learning (DL)-accelerated MRCP sequence (hereafter, MRCP_DL_) regarding acquisition time and image quality.

**Materials and methods:**

We conducted a prospective study of consecutive patients referred for MRCP between November 2023 and April 2024 at a single tertiary center. Each participant underwent 1.5T 3-D T2-weighted turbo spin echo MRCP using both a conventional sequence (threefold acceleration) and MRCP_DL_ (eightfold acceleration). Three blinded readers independently evaluated image quality, including background signal suppression, bile and pancreatic duct visibility, artifact level, and diagnostic confidence on an ordinal four-point scale. Acquisition times were compared using a paired t-test. Image quality parameters were assessed with repeated measures ANOVA. Interreader agreement was analyzed using Fleiss' κ.

**Results:**

Out of 419 consecutive patients, 30 participants were evaluated (mean age, 63 ± 15 years; 16 men, 14 women). The mean acquisition time was 10:30 ± 03:04 min for conventional MRCP and 3:57 ± 01:13 min for MRCP_DL_, *P* < 0.001. MRCP_DL_ reduced acquisition time by 62.4%. Artifact levels were rated at 3.17 ± 0.77 for conventional MRCP and 3.56 ± 0.66 for MRCP_DL_ (*P* = 0.041). Background signal suppression, bile duct visibility, pancreatic duct visibility, and diagnostic confidence did not differ significantly (*P* > 0.05). Interreader agreement was substantial to almost perfect (κ: 0.64–87).

**Conclusions:**

Deep learning-accelerated 3-D MRCP reduced acquisition time by 62%, minimized artifacts, and preserved bile and pancreatic duct visibility, supporting its adoption in routine clinical practice.

## Introduction

Magnetic Resonance Cholangiopancreatography (MRCP) is a non-invasive imaging technique that visualizes the bile and pancreatic ducts using heavy T2-weighting [1–3]. MRCP has become a crucial tool for diagnosing hepatobiliary and pancreatic disorders [4, 5]. Advancements in MRCP have led to the development of a free-breathing, three-dimensional (3-D) technique that improves patient comfort by eliminating the need for breath-holding, enables multiplanar reconstruction, and provides better duct visibility than 2-D MRCP [6–8]. Notably, 3-D MRCP entails longer acquisition times, which increases susceptibility to motion artifacts [9, 10]. Developments in respiratory triggering, such as Prospective Acquisition Correction (PACE), enable patients to breathe naturally during scans and have notably improved the quality of free-breathing 3-D MRCP [11]. Nevertheless, PACE-MRCP may still be subject to prolonged acquisition times when confronted with irregular breathing patterns.

Deep learning (DL) reconstruction methods have recently emerged as a promising approach to address these challenges. DL techniques are capable of reconstructing high-quality images from undersampled k-space data, thereby allowing for accelerated acquisition [12–15]. Reducing acquisition time can improve patient comfort, minimize motion artifacts, and optimize clinical efficiency. However, the application of DL reconstruction in MRCP is still a developing field. Its comparative performance with conventional MRCP techniques and its utility in real clinical settings remain underexplored, necessitating studies that bridge the gap between development and deployment.

We hypothesize that DL reconstruction of prospectively accelerated MRCP (MRCP_DL_) will significantly reduce scan time while preserving duct visibility and diagnostic confidence. Additionally, we anticipate that MRCP_DL_ may reduce artifact levels. Therefore, the purpose of our study was to compare a conventional 3-D MRCP sequence with a DL-reconstructed accelerated 3-D MRCP sequence (MRCP_DL_) in terms of acquisition time and image quality.

## Materials and methods

### Study design and participants

This prospective study was conducted at a single tertiary center (Tuebingen University Hospital, Tuebingen, Germany) after institutional review board approval. The study adhered to the Declaration of Helsinki and its amendments.

**Fig. 1:**
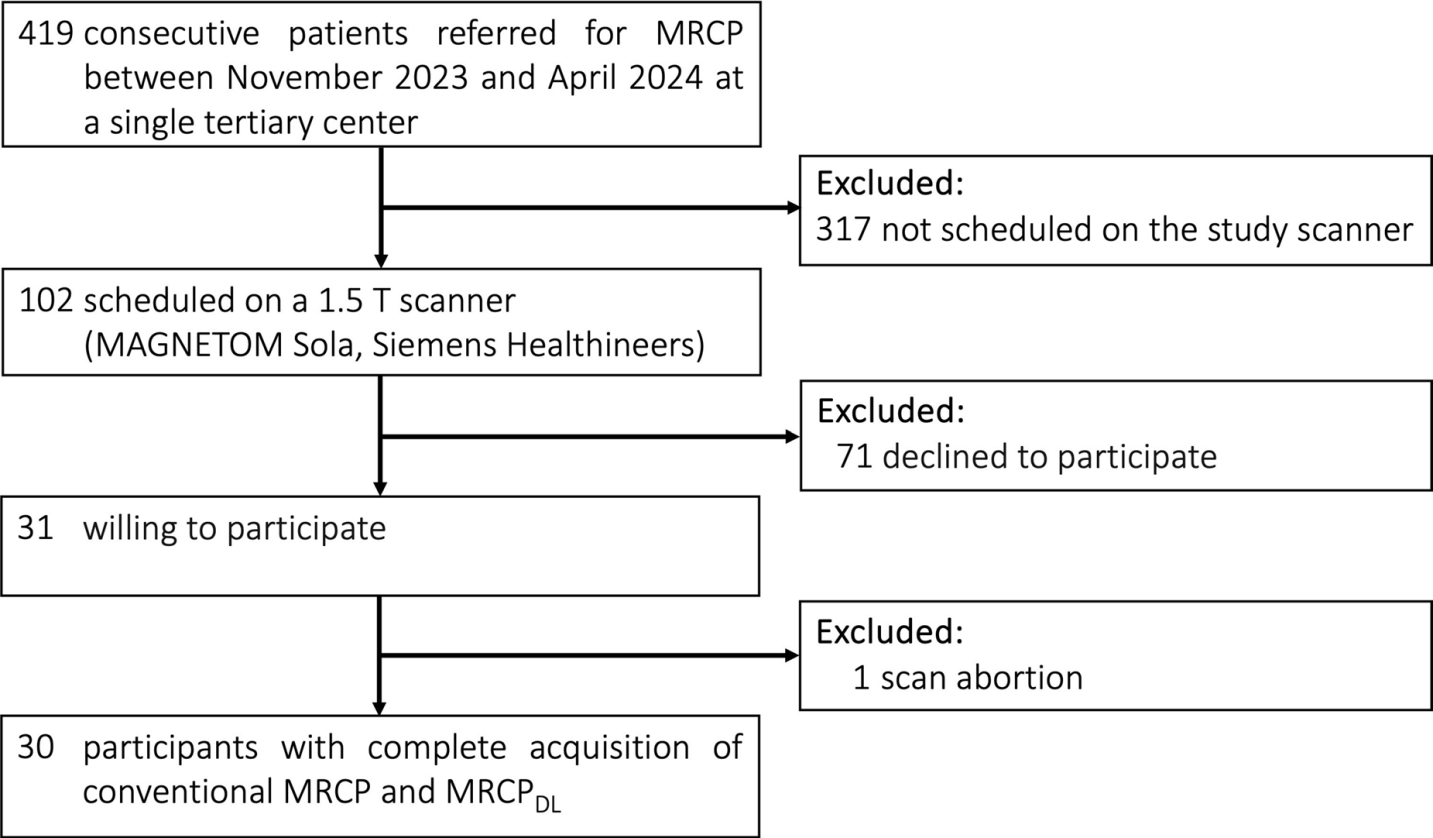
The flowchart illustrates participant inclusion and exclusion. DL, deep learning; MRCP, magnetic resonance cholangiopancreatography

Consecutive adult patients (≥ 18 years) referred for clinically indicated MRCP were enrolled between November 2023 and April 2024. Participation was voluntary. All participants gave written informed consent for the additional MRCP_DL_ sequence during the same examination. The MRCP_DL_ sequence was provided by Siemens Healthineers as a research package. The authors, who are unaffiliated with Siemens Healthineers, retained full control of the participants' data. Patients were excluded if they were not assigned to the study scanner, declined participation, or had incomplete sequence acquisition. Baseline demographics, referring departments, and reasons for referral are shown in Fig. 1 and Table 1.

**Table 1:** Baseline characteristics

Characteristic	Value (*n* = 30)
Demographics	
Mean age (years)	63 ± 15 [20–85]
Median age (years)	65 (54, 74)
Women	14 (14/30, 45.2)
Men	16 (16/30, 54.8)
Referring department	
Oncology	11 (11/30, 36.7)
Gastroenterology	10 (10/30, 33.3)
Transplant surgery	5 (5/30, 16.7)
Visceral surgery	3 (3/30, 10.0)
Hepatology	1 (1/30, 3.3)
Reason for referral	
Intraductal papillary mucinous neoplasm	8 (8/30, 26.7)
Cholestasis	4 (4/30, 13.3)
Cholangiocellular carcinoma	2 (2/30, 6.7)
Cholangitis	2 (2/30, 6.7)
Concern for pancreatic cancer	2 (2/30, 6.7)
Pancreatitis	2 (2/30, 6.7)
Primary sclerosing cholangitis	2 (2/30, 6.7)
Autoimmune hepatitis	1 (1/30, 3.3)
Concern for cholangiocellular carcinoma	1 (1/30, 3.3)
Concern for gall bladder cancer	1 (1/30, 3.3)
Gall bladder polyp	1 (1/30, 3.3)
Pancreatic neuroendocrine tumor	1 (1/30, 3.3)
Pancreatic carcinoma	1 (1/30, 3.3)
Pancreatic cystadenoma	1 (1/30, 3.3)
Primary biliary cholangitis	1 (1/30, 3.3)

Quantitative variables are presented as mean ± standard deviation with ranges in brackets, or as median with first and third quartiles in parentheses (Q1, Q3). Qualitative variables are reported as raw numbers, with proportions and percentages in parentheses

### MRI protocol

Magnetic resonance imaging (MRI) examinations were performed using a 1.5-T scanner (MAGNETOM Sola, Siemens Healthineers). Each participant underwent 3-D MRCP using a T2-weighted turbo spin-echo sequence (Sampling Perfection with Application optimized Contrast using different flip angle Evolution, SPACE) [16, 17] with threefold GRAPPA (GeneRalized Autocalibrating Partial Parallel Acquisition) acceleration and a MRCP_DL_ research sequence with eigthfold CAIPIRINHA (Controlled Aliasing in Parallel Imaging Results in Higher Acceleration) acceleration. The sequences were acquired during free breathing with automatic PACE (Prospective Acquisition Correction) triggering for motion management. Both sequences captured ninety-six slices in coronal-oblique orientation, each with a thickness of 1.0 mm. The detailed MRI acquisition parameters are provided in Table 2.

**Table 2:** Acquisition parameters at 1.5T

Parameter	MRCP	MRCP_DL_
Sequence	3-D T2-weighted TSE (SPACE)	3-D T2-weighted TSE (SPACE)
Slice thickness [mm]	1.0	1.0
Number of slices	96	96
Matrix	384 × 384	384 × 384
Field of view [mm^2^]	400 × 400	400 × 400
Voxel size [mm^3^]	1.0 × 1.0 × 1.0	1.0 × 1.0 × 1.0
Acceleration mode	GRAPPA	CAIPIRINHA
Total acceleration factor	3	8
Acceleration factor PE	3	4
Acceleration factor 3D	1	2
Slice partial Fourier	7/8	6/8
Repetition time [ms]	2400.0	2400.0
Echo time [ms]	695.00	425.00
Echo Train Duration [ms]	1133	809
Echo trains (PACE training + data acquisition)	5 + 126	5 + 52
Turbo factor	240	180
Flip angle [degree]	140	120

CAIPIRINHA, Controlled Aliasing in Parallel Imaging Results in Higher Acceleration; DL, deep learning; GRAPPA, GeneRalized Autocalibrating Partial Parallel Acquisition; PACE, Prospective Acquisition Correction; PE, phase-encoding

### DL reconstruction technique

**Fig. 2:**
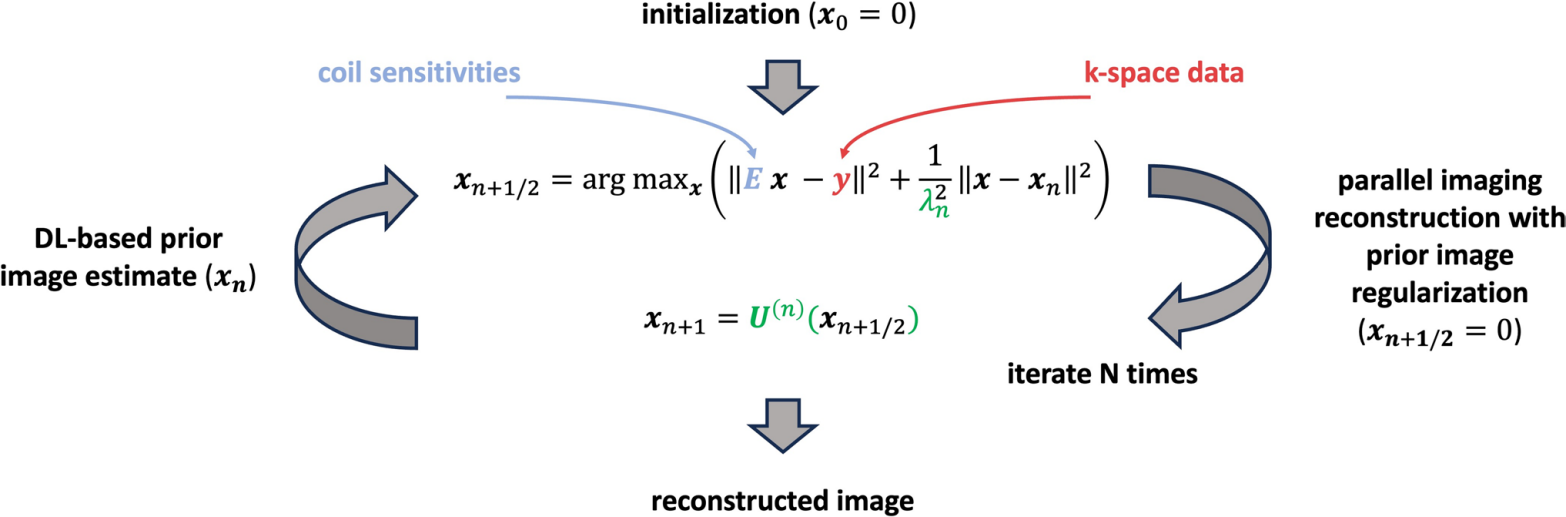
Illustration of deep learning-based image reconstruction. The algorithm performs a fixed number of iterations alternating between conventional parallel imaging with an image prior and neural network based image regularization. Conventional image reconstruction includes a data consistency term that relates the current image estimate to the acquired k-space data using coil sensitivity maps, as well as l2 regularization that refers to a given image prior. The output of this parallel imaging step is then fed into a neural network to estimate the image prior for the next iteration. The parameters of the neural networks and the regularization strengths of the l2 regularizations are determined through supervised training

The DL-based image reconstruction comprised a variational network [14], as previously used for abdominal T1-weighted imaging [18–20], and is shown in Fig. 2. The algorithms receive the acquired k-space data and precalculated coil sensitivity maps to generate the DL-enhanced images by alternating between parallel imaging-based data consistency and neural network-based regularization. The overall architecture was trained offline on a dedicated GPU server end-to-end in a supervised fashion using 500 fully sampled datasets obtained from healthy volunteers in various body regions on 1.5T and 3T scanners (MAGNETOM scanners, Siemens Healthineers). The obtained parameters were then exported for prospective use in the scanner integrated reconstruction pipeline.

### Image analysis

The image analysis was conducted by three independent readers. S.G., a board-certified radiologist, has 6 years of experience in abdominal MRI interpretation. J.M.B. and R.D. are residents, each with 2 years of experience. Prior to initiating the analysis, all readers underwent training in image interpretation and scoring. The readers were blinded to the type of acquisition, with all participant and sequence identifiers removed from the images. To mitigate the potential for recall bias, each reader analyzed the two datasets (conventional MRCP and MRCP_DL_) in separate sessions. The order of the analyses was randomized and mixed, with a minimum 4-week interval between sessions to allow for sufficient washout. The readers did not have access to the evaluations performed by their colleagues. Readers assessed image quality by evaluating background signal suppression, bile and pancreatic duct visibility (including the common bile duct, right and left 1st- and 2nd-order intrahepatic ducts, and for the pancreas, the head, body, and tail segments), artifact levels, and diagnostic confidence. These aspects were rated on a four-point scale (1 = poor, 2 = moderate, 3 = good, 4 = excellent), with higher ratings indicating better image quality. Higher scores reflected enhanced background signal suppression, clearer duct visualization, fewer artifacts, and elevated diagnostic confidence. Analysis was carried out on a dedicated workstation (GE Centricity PACS RA1000; GE HealthCare) within a certified reading room.

### Statistical analysis

The acquisition times for conventional MRCP and MRCP_DL_ were compared using a paired t-test. Image quality parameters were analyzed by repeated measures analysis of variance (ANOVA). Interobserver agreement was assessed using Fleiss' Kappa [21] and categorized as follows: 0.00–0.20 = slight; 0.21–0.40 = fair; 0.41–0.60 = moderate; 0.61–0.80 = substantial; 0.81–1.00 = almost perfect. The first author (J.M.B.) performed the statistical analysis using SPSS v29.0 (IBM). Continuous data are presented as means ± standard deviations or medians with interquartile ranges. Categorical data are presented as proportions and percentages. A two-tailed P-value of less than 0.05 was considered statistically significant.

## Results

### Baseline participant characteristics

**Fig. 3:**
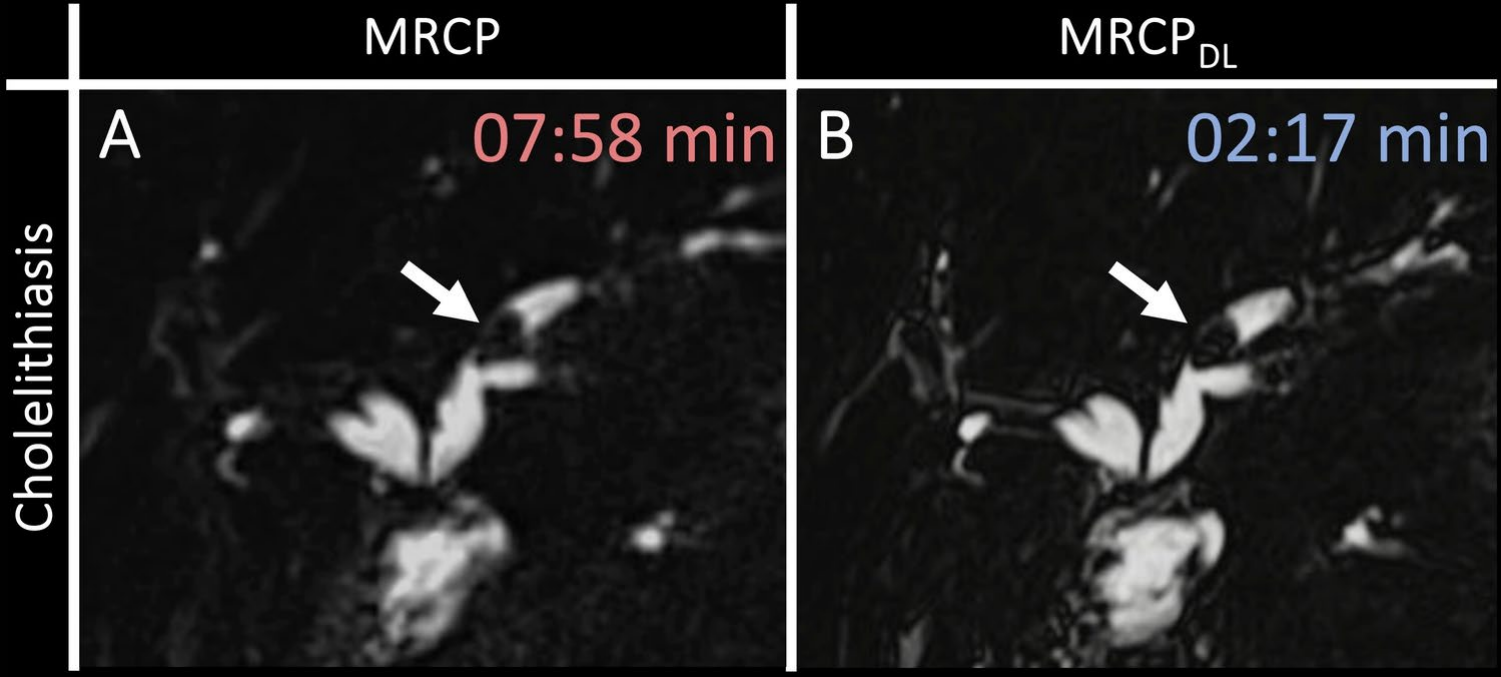
Magnetic resonance cholangiopancreatography (MRCP) of a 64-year-old woman, referred for suspected cholelithiasis during a post-liver transplant check-up. Both **A** MRCP and **B** MRCP_DL_ clearly depict the stone in the left bile duct (white arrows). Both sequences show comparable overall image quality. The MRCP acquisition time was 07:58 min, while MRCP_DL_ took 02:17 min. DL, deep learning; MRCP, magnetic resonance cholangiopancreatography

Among the 419 consecutive patients referred for MRCP (Fig. 1), 317 were excluded due to being assigned to different scanners than the study scanner, which featured the DL reconstruction algorithm. Additionally, 71 patients were excluded because they declined participation. From the remaining 31 patients who consented and were scanned, one MRI dataset was incomplete due to scan abortion. Consequently, 30 participants completed the clinically indicated 1.5T MRCP with full study sequence acquisition. Participants had a mean age of 63 ± 15 years [standard deviation, SD] (median 65 years [IQR, 54–74]; range, 20–85 years), comprising 16 men and 14 women. Table 1 details participant baseline characteristics including demographics, referring departments, and reasons for referral. Most participants were referred from oncology (11/30, 36.7%), followed by gastroenterology (10/30, 33.3%) and transplant surgery (5/30, 16.7%). The most common reason for referral was intraductal papillary mucinous neoplasm (IPMN) of the pancreas (8/30, 26.7%), followed by cholestasis (4/30, 13.3%). Cholangiocellular carcinoma, cholangitis, concern for pancreatic cancer, pancreatitis, and primary sclerosing cholangitis each accounted for 2/30 cases (6.7%). Figure 3 showcases example images of conventional MRCP and MRCP_DL_ from a patient with cholelithiasis.

### Comparison of acquisition time

The mean acquisition time for conventional MRCP was 10:30 ± 03:04 min compared to 3:57 min ± 01:13 min for MRCP_DL_, *P* < 0.001. The acquisition time ranged from 06:30 to 17:10 min for conventional MRCP and from 02:02 to 06:47 min for MRCP_DL_. Compared to conventional MRCP, MRCP_DL_ showed a 62.4% decrease in mean acquisition time.

### Image quality of conventional MRCP and MRCP_DL_

**Fig. 4:**
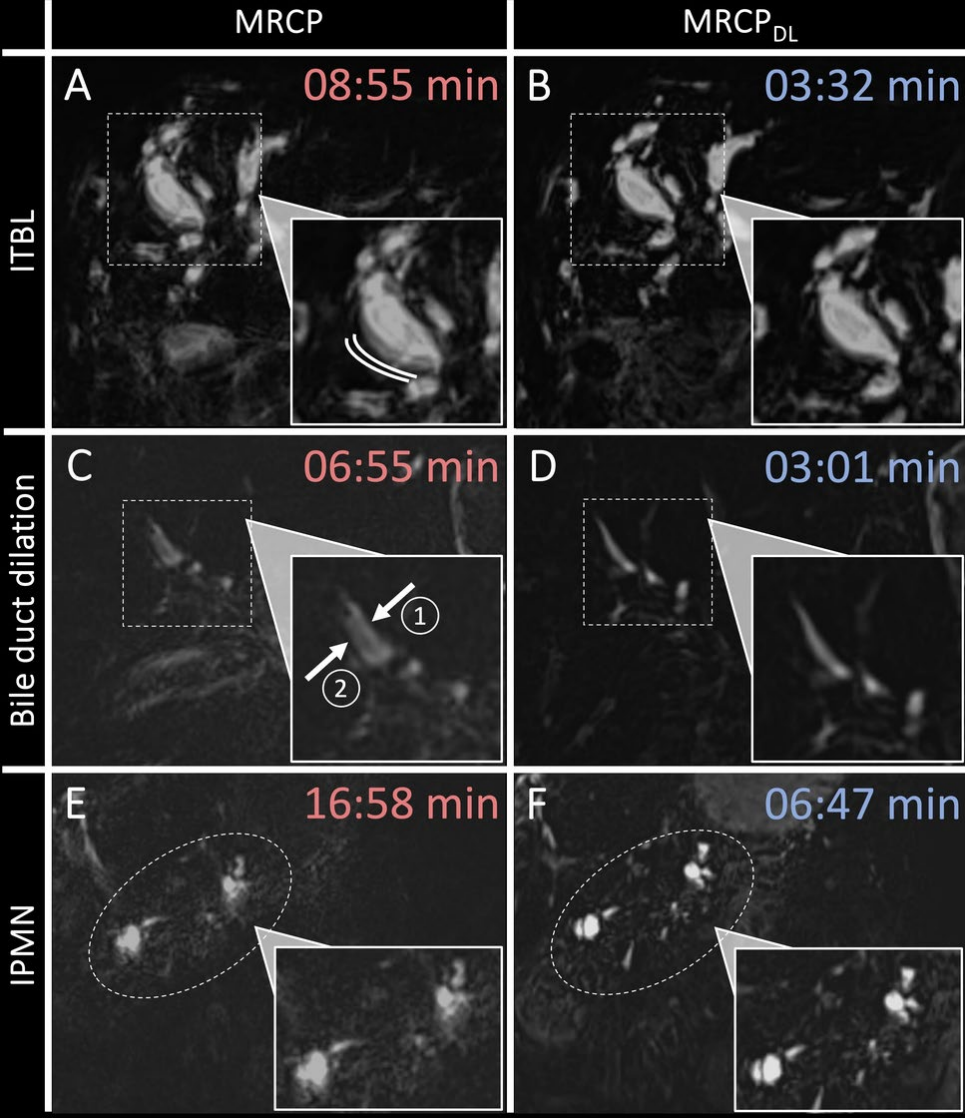
Top row: A 62-year-old patient with ischemic-type biliary lesions (ITBL) after a liver transplant 22 years ago underwent magnetic resonance cholangiopancreatography (MRCP). **A** The conventional MRCP shows motion artifacts, evident as double wall depiction (white lines in A), **B** while the MRCP_DL_ delineates the bile duct wall without artifacts. Middle row: MRCP in a 67-year-old patient showing mild intrahepatic bile duct dilation due to a juxtapapillary duodenal diverticulum. **C** Conventional MRCP shows motion artifacts, indicated by double depiction of a right-sided intrahepatic bile duct (arrows one and two). **D** MRCP_DL_ reveals no motion artifacts and provides a clear visualization of the intrahepatic bile duct. Bottom row: MRCP was performed on a 72-year-old patient for follow-up of intraductal papillary mucinous neoplasms (IPMNs) in the pancreas. **E** Conventional MRCP, which required 16:58 min, shows the IPMNs with blurring. **F** MRCP_DL_, taking 06:47 min, provides a clearer depiction of the IPMNs. Overall, note the substantially shorter acquisition times for MRCP_DL_ compared to conventional MRCP. DL, deep learning; IPMN, intraductal papillary mucinous neoplasm; ITBL, ischemic-type biliary lesions; MRCP, magnetic resonance cholangiopancreatography

Table 3 summarizes the image quality assessment results. The mean score for background signal suppression was 3.13 ± 0.69 for conventional MRCP and 3.16 ± 0.65 for MRCP_DL_, *P* = 0.898. Bile duct visibility did not differ significantly between conventional MRCP and MRCP_DL_, *P* > 0.05 for all segments. Pancreatic duct visibility did not differ significantly between conventional MRCP and MRCP_DL_, *P* > 0.05 for all segments. The artifact level was rated 3.17 ± 0.77 for conventional MRCP and 3.56 ± 0.66 for MRCP_DL_, *P* = 0.041. Figure 4 shows MRCP examples highlighting differences in artifact levels between the two sequences. Diagnostic confidence was rated 3.29 ± 0.73 for conventional MRCP and 3.41 ± 0.67 for MRCP_DL_, *P* = 0.501.

**Table 3:** Image quality of MRCP and MRCP_DL_

Parameter	MRCP		MRCP_DL_		*P* value
	Rating	Agreement	Rating	Agreement	
Background signal suppression	3.13 ± 0.69 (2–4)	0.72	3.16 ± 0.65 (2–4)	0.64	0.898
Bile duct visibility					
Common bile duct	3.58 ± 0.53 (2–4)	0.78	3.66 ± 0.54 (2–4)	0.80	0.577
Right hepatic duct (1st)	3.51 ± 0.59 (2–4)	0.75	3.57 ± 0.65 (2–4)	0.77	0.731
Right hepatic duct (2nd)	3.29 ± 0.64 (2–4)	0.70	3.36 ± 0.62 (2–4)	0.73	0.684
Left hepatic duct (1st)	3.44 ± 0.61 (2–4)	0.76	3.54 ± 0.59 (2–4)	0.74	0.521
Left hepatic duct (2nd)	3.18 ± 0.65 (2–4)	0.67	3.31 ± 0.66 (2–4)	0.71	0.435
Pancreatic duct visibility					
Head	3.40 ± 0.73 (2–4)	0.76	3.47 ± 0.66 (2–4)	0.79	0.707
Body	3.39 ± 0.65 (2–4)	0.69	3.41 ± 0.65 (2–4)	0.68	0.893
Tail	3.36 ± 0.73 (2–4)	0.73	3.45 ± 0.70 (2–4)	0.71	0.627
Artifact level	3.17 ± 0.77 (2–4)	0.86	3.56 ± 0.66 (2–4)	0.87	0.041
Diagnostic confidence	3.29 ± 0.73 (2–4)	0.75	3.41 ± 0.67 (2–4)	0.85	0.501

Data are presented as mean ± standard deviation (range). Interobserver agreement for the three readers is given as Fleiss' Kappa, categorized as follows: 0.00–0.20 = slight; 0.21–0.40 = fair; 0.41–0.60 = moderate; 0.61–0.80 = substantial; 0.81–1.00 = almost perfect

DL, deep learning; MRCP, magnetic resonance cholangiopancreatography

### Interreader agreement (κ statistics)

The interreader agreement for image quality parameters in conventional MRCP varied from substantial to almost perfect, with κ-values from 0.67 to 0.86, Table 3. For MRCP_DL_, interreader agreement ranged from substantial to almost perfect as well, with κ-values between 0.64 and 0.87.

## Discussion

This study compared a conventional three-dimensional (3-D) magnetic resonance cholangiopancreatography (MRCP) sequence with a deep learning (DL) reconstructed accelerated 3-D MRCP sequence (MRCP_DL_), focusing on acquisition time and image quality. The findings demonstrate a significant reduction in acquisition time with MRCP_DL_, decreasing from an average of 10:30 min to 3:57 min, representing a 62.4% reduction (*P* < 0.001). Notably, while MRCP_DL_ substantially accelerated the imaging process, it did so without compromising image quality. These results indicate that MRCP_DL_ offers a considerable advantage in reducing scan time while maintaining diagnostic accuracy, thereby potentially enhancing clinical efficiency and patient throughput.

The study’s findings align with recent studies that demonstrate the superiority of DL-reconstructed acquisitions over conventional methods in reducing acquisition time while maintaining or enhancing image quality in various areas of abdominal imaging [19, 20, 22–27]. For 3-D MRCP specifically, efforts to reduce the traditionally long acquisition times have been ongoing for years [28]. Techniques such as compressed sensing and parallel imaging have achieved reductions in scan time for 3-D MRCP of up to 50% [7, 28]. However, deep learning models have been shown to outperform these techniques in maintaining image quality while significantly reducing acquisition times [29]. An important consideration in our study is the substantially shorter echo time (TE) utilized in the DL-based MRCP sequence (TE = 435 ms) compared to the conventional MRCP (TE = 695 ms). Shorter TE increases signal contributions from tissues with shorter T2 relaxation times, leading to elevated background signal and potentially diminishing the contrast between biliary structures and surrounding tissues. Despite this inherent challenge, our findings demonstrated that the background signal suppression of the DL-based MRCP was comparable to that of the conventional sequence. Specifically, the mean scores for background signal suppression were 3.13 ± 0.69 for conventional MRCP and 3.16 ± 0.65 for DL-based MRCP (*P* > 0.05). This suggests that the advanced DL reconstruction algorithm effectively compensates for the increased background signal associated with the shorter TE, enhancing background suppression. The ability of the DL-based reconstruction to maintain effective background suppression despite a shorter TE may contribute to improved image quality and diagnostic confidence in MRCP examinations. Maintaining diagnostic yield is essential, and the focus should be on retaining the acquisition of MRCP sequences beneficial for disease detection, particularly given the complex ductal anatomy. With particular emphasis on this, our study showed no significant differences in duct visibility between conventional MRCP and MRCP_DL_. Bile duct visibility and pancreatic duct visibility yielded mean ratings between 3.18 ± 0.65 and 3.58 ± 0.53, for conventional MRCP, compared to mean ratings between 3.31 ± 0.66 and 3.66 ± 0.54 for MRCP_DL_ (all *P* > 0.05). These findings underscore the efficacy of deep neural networks in generating high-quality images despite substantial undersampling of k-space data [30]. In cases of irregular breathing patterns and prolonged acquisition times despite the implementation of MRCP_DL_, a breath-hold 3-D MRCP may be considered as an alternative [31]. However, this approach entails a trade-off between the increased susceptibility to motion artifacts in respiratory-triggered 3-D MRCP and the diminished spatial resolution resulting from the constrained time window in breath-hold 3-D MRCP. Recent developments in deep learning reconstruction may facilitate enhanced image quality even in patients with irregular breathing patterns [32]. Regarding artifacts, our study showed encouraging results, with lower artifact levels in MRCP_DL_ compared to conventional MRCP (3.56 ± 0.66 vs. 3.17 ± 0.77, *P* = 0.041), likely due to the shorter acquisition times observed with MRCP_DL_. Overall, deep learning reconstruction for accelerated MRCP demonstrated robust performance. Future studies should be encouraged to conduct cost–benefit analyses evaluating the economic impact of implementing accelerated MRCP_DL_ in clinical practice with regard to potential savings from reduced scan times and improved workflow efficiency. Quicker patient throughput may allow clinicians to make more timely diagnoses and initiate appropriate treatments without unnecessary delays. Additionally, patient-centered research may investigate the impact of this technique on patient experience, particularly in terms of comfort and overall well-being during the scanning process, contributing to a more efficient and patient-friendly clinical environment. Overall, the adoption of deep learning-based accelerated MRCP has the potential to enhance both the quality of diagnostic information and the effectiveness of patient care.

There are limitations to this study. First, the study was performed at a single tertiary center with a limited sample size, potentially restricting the generalizability of the findings to other settings, equipment and patient populations. Second, although standardized criteria were employed, image quality assessment was subjective, relying on individual reader interpretation. Variability among radiologists performing image analysis may lead to inconsistencies in evaluating image quality, duct visibility, and diagnostic confidence. Nonetheless, a quantitative approach is subject to its own limitations. In this study, the MRI sequences were acquired prospectively rather than being retrospectively reconstructed from conventional images. Consequently, direct comparison of quantitative measurements is precluded due to differences in organ and duct orientation or configuration caused by physiological factors such as respiratory depth and patient movement between sequence acquisitions. Third, whilst the study demonstrates a reduction in acquisition time and the preservation of image quality, additional research is required to ascertain the broader clinical impact on patient care and management, extending beyond the immediate image-based observations.

In conclusion, deep learning reconstruction for accelerated 3-D MRCP reduced acquisition time by 62%, minimized artifacts, and preserved the visibility of bile and pancreatic ducts. These findings support its integration into routine clinical practice. Incorporating this approach into standard MRCP and abdominal MRI protocols may improve patient comfort and increase throughput.

## Abbreviations

3-D: Three-dimensional
DL: Deep learning
MRCP: Magnetic resonance cholangiopancreatography
PACE: Prospective acquisition correction
SPACE: Sampling Perfection with Application optimized Contrast using different flip angle Evolution
